# Prefrontal cortex neuronal ensembles encoding fear drive fear expression during long-term memory retrieval

**DOI:** 10.1038/s41598-019-47095-7

**Published:** 2019-07-24

**Authors:** Giuseppe Giannotti, Jasper A. Heinsbroek, Alexander J. Yue, Karl Deisseroth, Jamie Peters

**Affiliations:** 1 Department of Neuroscience, Medical University of South Carolina, Charleston, SC USA; 2 Department of Anesthesiology, Department of Pharmacology, University of Colorado Denver, Anschutz Medical Campus, Aurora, CO USA; 3 Department of Bioengineering, CNC Program, Stanford University, Stanford, CA USA; 4 Department of Psychiatry, Department of Biology, Stanford University, Stanford, CA USA

**Keywords:** Fear conditioning, Long-term memory

## Abstract

The prefrontal cortex is an important regulator of fear expression in humans and rodents. Specifically, the rodent prelimbic (PL) prefrontal cortex drives fear expression during both encoding and retrieval of fear memory. Neuronal ensembles have been proposed to function as memory encoding units, and their re-activation is thought to be necessary for memory retrieval and expression of conditioned behavior. However, it remains unclear whether PL cortex neuronal ensembles that encode fear memory contribute to long-term fear expression during memory retrieval. To address this, we employed a viral-mediated TRAP (Targeted Recombination in Active Population) technology to target PL cortex ensembles active during fear conditioning and expressed the inhibitory Gi-DREADD in fear-encoding ensembles. Male and female rats were trained to lever press for food and subjected to Pavlovian delay fear conditioning, then 28 days later, they underwent a fear memory retrieval test. Chemogenetic inhibition of TRAPed PL cortex ensembles reduced conditioned suppression of food seeking in females, but not males. Neither context nor tone freezing behavior was altered by this manipulation during the same retrieval test. Thus, fear-encoding ensembles in PL cortex drive long-term fear expression in a sex and fear modality dependent manner.

## Introduction

Fear disorders, including anxiety and post-traumatic stress disorder (PTSD), are characterized by an exacerbated fear response, as well as impaired extinction learning and memory^1,2^. Neuroimaging techniques have been critical in identifying the neural circuits impacted by PTSD, and several studies indicate that the dorsal anterior cingulate cortex is hyperactivated by fearful stimuli^3^. The rodent equivalent of the dorsal anterior cingulate cortex, the prelimbic (PL) prefrontal cortex, is active during acquisition (i.e. encoding) of fear memories and has been implicated in consolidation of the original fear memory^4^. For instance, PL cortex neurons increase firing during tone presentations over the course of fear conditioning (FC), as animals learn to associate the tone with impending shock^5^, and PL neuron tone responses correlate with tone freezing during FC^6^. Moreover, PL cortex activity drives fear expression during both acquisition (i.e. fear encoding) and memory retrieval^7,8^. Electrical microstimulation of PL cortex increases conditioned tone freezing during memory retrieval^9^, and disinhibition of PL cortex pyramidal neurons by inhibition of parvalbumin interneurons induces fear expression^10^. Pharmacological inactivation studies indicate that PL cortex activity is necessary for expression of conditioned tone freezing during both fear memory acquisition and retrieval, but PL cortex activity during FC is not necessary for fear expression during retrieval^7^, suggesting that the neural substrates underlying encoding versus retrieval in PL cortex may be distinct. In line with this, the outputs by which PL cortex drives fear expression change over time^11^. Few studies have directly addressed whether the same PL cortex neurons that encode fear memory contribute to fear expression during memory retrieval.

According to the engram theory of memory^12–14^, acquisition of memories and memory storage is an active neurobiological process during which a small population of neurons (ensemble or engram) is activated and undergoes persistent physical and chemical changes. Subsequently, when the stimulus returns, these neurons are reactivated to evoke the recall of specific memories^12^. However, due to the lack of temporal resolution and methodological limitations, it is still unknown how specific neurons that are active during acquisition of memories contribute to memory recall^15^. Different strategies and animal models are available to address this question and obtain genetic access to neuronal ensembles activated by specific stimuli^16,17^. TRAP (Targeted Recombination in Active Populations) allows genetic access to active neurons with precise temporal resolution, using a Fos promoter to drive expression of CreER^T2^ recombinase only when 4-hydroxytamoxifen (4-OHT) is systemically administered at the time of interest^18^. Moreover, the TRAP technology can be applied using TRAP transgenic mice, or by using a viral vector (i.e. AAV) in wildtype animals to deliver the Fos-CreER^T2^ ^18,19^. A recent study using TRAP transgenic mice found marginal effects of manipulating fear-encoding ensembles on long-term memory retrieval^20^. Thus, more work is needed to address the conditions under which this population controls fear expression at remote memory time points.

To fill this gap in knowledge, we employed the AAV-TRAP technology in rats to investigate whether PL cortex neuronal ensembles active during fear memory encoding are required for long-term fear memory retrieval. We TRAPed the inhibitory hM4Di designer receptor (Gi-DREADD) into a fear memory-encoding PL cortex ensemble by administering 4-OHT to male and female rats after a FC session. We analyzed two different behavioral measures of fear during remote memory retrieval (28 d), freezing and conditioned suppression of food seeking.

## Results

### Validation of viral TRAP strategy in rats

**Figure 1:**
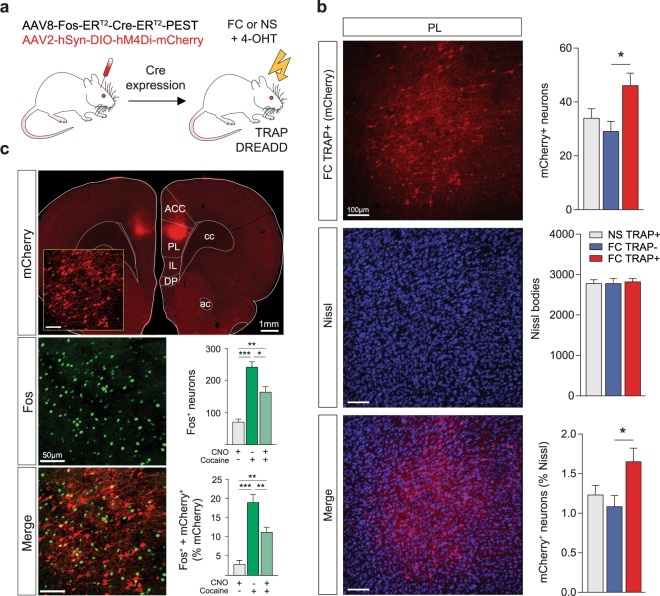
Validation of viral mediated TRAP technology in rats. The TRAP-DREADD methodology (**a**) involved the co-injection of two viral vectors into the PL cortex of wildtype male and female rats. After allowing the inducible Cre to express, PL ensembles were TRAPped with the Gi-DREADD after fear conditioning (FC) by systemic injection of 4-OHT. No shock (NS) controls were used to TRAP a no-fear ensemble. Validation of the induction efficiency of the TRAP-Gi-DREADD in PL cortex (**b**) across experimental groups expressed as total mCherry^+^ neurons and % Nissl^+^ neurons. mCherry (red) = Gi-DREADD, blue = Nissl, purple = co-labeled. The inhibitory function of the TRAP-Gi- DREADD (**c**) was verified by examining the ability of CNO to reduce Fos expression induced by cocaine. mCherry (red) = Gi-DREADD, green = Fos, yellow = co-labeled. *p < 0.05, **p < 0.01, ***p < 0.001 Tukey’s post-hocs.

Figure 1a depicts the viral strategy used in our experiments. The PL cortex of wild-type male and female rats was injected with the TRAP vector (AAV8-Fos-ER^T2^-Cre-ER^T2^-PEST) and the Cre-dependent Gi-DREADD (AAV2-hSyn-DIO-hM4Di-mCherry). At the conclusion of the experiment, we validated the induction efficiency of the TRAP-Gi-DREADD in PL cortex. Figure 1b shows representative images of Gi-DREADD expression (mCherry, red), overall neuronal population (Nissl, blue), and co-localization of these markers (purple). Statistical analysis of total mCherry+ neurons revealed a significant induction of TRAP-Gi-DREADD in animals that underwent FC and injection with 4-OHT (FC TRAP+) compared to those injected with vehicle (FC TRAP−) (One-Way ANOVA: F_(2,21)_ = 4.621, *p* = 0.022; post-hoc FC TRAP− vs. FC TRAP+ *p* = 0.019). No statistical differences were observed in the number of Nissl bodies between groups. Moreover, we found a similar induction efficiency of the TRAP-Gi-DREADD when the number of mCherry+ neurons was normalized as a percentage of Nissl bodies (One-Way ANOVA: F_(2,21)_ = 3.938, *p* = 0.035; post-hoc FC TRAP+ vs. FC TRAP− *p* = 0.033).

### Verification of TRAP-Gi-DREADD functionality

To validate the inhibitory activity of the TRAP-Gi-DREADD, at the end of the experiment we injected a subset of rats with cocaine to induce Fos expression and examined the efficacy of CNO in mitigating Fos induction. Figure 1c (top left) shows the TRAP-Gi-DREADD expression in PL cortex (mCherry, red), Fos induction (green), and the co-localization of Fos in the TRAPed ensemble (yellow). As expected, cocaine induced Fos expression in the overall neuronal population, but this was mitigated by pretreatment with CNO (One-Way ANOVA: F_(2,21)_ = 13.03, *p* < 0.001; post-hocs CNO+/cocaine− vs. CNO−/cocaine+: *p* < 0.001, CNO+/cocaine− vs. CNO+/cocaine+: *p* = 0.008, CNO−/cocaine+ vs. CNO+/cocaine+: *p* = 0.027). Furthermore, this effect was attributed to the TRAPed ensemble containing the Gi-DREADD (Fos+ neurons as % mCherry+) (One-Way ANOVA: F_(2,21)_ = 15.87, *p* < 0.001; post-hocs CNO+/cocaine− vs. CNO−/cocaine+: *p* < 0.001, CNO+/cocaine− vs. CNO+/cocaine+: *p* = 0.005, CNO−/cocaine+ vs. CNO+/cocaine+: *p* = 0.009).

### Prelimbic cortex ensembles encoding fear regulate fear expression

**Figure 2:**
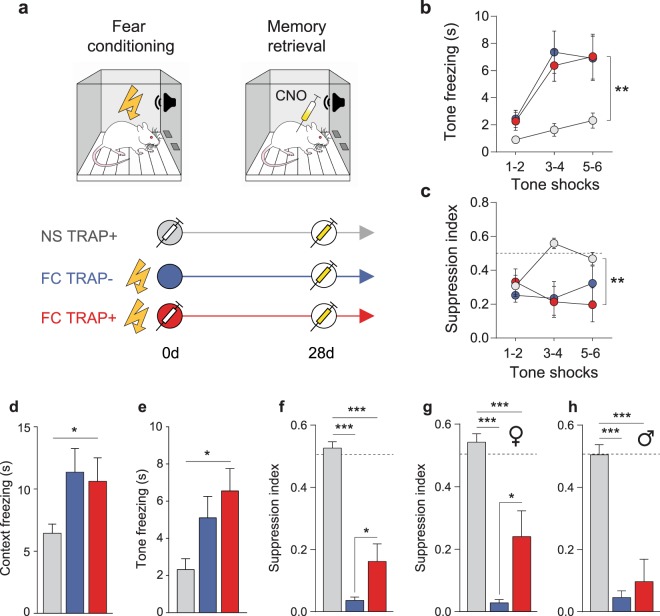
Prelimbic cortex ensembles encoding fear regulate fear expression. The experimental timeline and groups for behavioral experiments (**a**) included fear conditioning (FC) (or no-shock controls, NS) followed by TRAP+ (4-OHT) or TRAP- (vehicle) injections, and 28 d later, a memory retrieval test with CNO. Freezing (**b**) and conditioned suppression of food seeking (**c**) were measured as indices of conditioned fear. Freezing to context (**d**) and tone (**e**) as well as suppression of food seeking (**g**) were measured after ensemble-inhibition on the d 28 memory retrieval test. The fear-reducing effect of the TRAP-Gi-DREADD on suppression index was apparent only in females (**g**), not males (**h**). *p < 0.05, **p < 0.01 treatment effect (**b**–**e**); *p < 0.05, **p < 0.01, ***p < 0.001 Tukey’s post-hocs (**e**–**h**).

Figure 2a depicts the experimental procedure (top) and the experimental groups (bottom). Male and female rats were randomly assigned to 2 different groups in equal number: (1) a control group that did not receive shock (NS) and (2) a group that underwent a FC session in which 6 tones co-terminated with an electric footshock. To target neuronal ensembles active during fear memory encoding, FC rats were injected with 4-OHT (FC TRAP+). A third group received vehicle instead of 4-OHT (FC TRAP−). NS controls received 4-OHT (NS TRAP+) to verify the specificity of any ensemble effects on fear memory. We analyzed two behavioral measures of fear, freezing and conditioned suppression of food seeking; the latter is a more sensitive measure of fear^21^. Conditioned tone freezing in FC groups was greater compared to NS controls during FC (Fig. 2b) (Two-Way RM ANOVA: treatment F_(2,33)_ = 7.384, *p* = 0.002; trial F_(2,66)_ = 24.04, *p* < 0.001; interaction F_(4,66)_ = 3.261, *p* = 0.017), as was conditioned suppression of food seeking (Fig. 2c) (Two-Way RM ANOVA: treatment F_(2,33)_ = 7.137, *p* = 0.003).

Twenty-eight days after the FC session, rats underwent a memory retrieval test. CNO was administered 30 min prior to the start of the test to inhibit FC ensembles in PL cortex. As expected, there was an effect of treatment on freezing to context (Fig. 2d) (One-Way ANOVA: F_(2,33)_ = 3.332, *p* = 0.048) and tones (Fig. 2e) (One-Way ANOVA: F_(2,33)_ = 5.231, *p* = 0.011). Tone freezing was somewhat more pronounced after chemogenetic inhibition of PL ensembles (post-hoc NS TRAP+ vs. FC TRAP+: *p* = 0.010), although there was no statistical difference between FC TRAP groups. There was also an effect of treatment on suppression of food seeking (Fig. 2f) (One-Way ANOVA: F_(2,33)_ = 62.16, *p* < 0.001). Chemogenetic inhibition of PL ensembles attenuated conditioned suppression of food seeking (post-hocs NS TRAP+ vs. FC TRAP+: *p* < 0.001; NS TRAP+ vs. FC TRAP−: *p* < 0.001; FC TRAP+ vs. FC TRAP−: *p* = 0.040). Because treatment groups were balanced with males and females, we examined these data separately by sex. Interestingly, the effect on suppression index could be attributed exclusively to females (Fig. 2g) (One-Way ANOVA: F_(2,16)_ = 41.38, *p* < 0.001; NS TRAP+ vs. FC TRAP−: *p* < 0.001, NS TRAP+ vs. FC TRAP+: *p* < 0.001, FC TRAP+ vs. FC TRAP−: *p* = 0.012) and was not observed in males (Fig. 2h) (One-Way ANOVA: F_(2,14)_ = 26.05, *p* < 0.001; NS TRAP+ vs. FC TRAP+: *p* < 0.001). No sex differences were observed in context or tone freezing on this test (Supplemental Fig. S1).

## Discussion

In this study we validated and employed a viral mediated TRAP strategy to investigate the contribution of fear-encoding (FC) ensembles on long-term memory retrieval. Chemogenetic inhibition of PL cortex FC ensembles during remote (28 d) memory retrieval reduced fear expression measured by conditioned suppression of food seeking but had no effect on conditioned freezing to context or tones. Our findings suggest that the PL ensembles encoding fear during memory acquisition play a critical role in distinct aspects of fear expression during later retrieval. Moreover, the fear-encoding ensemble participated in long-term memory retrieval only in female rats, highlighting the importance of investigating both sexes in fear and stress related studies.

PL cortex neuronal activity is required for fear memory expression during conditioning^22^ and later when the fear memory is retrieved after the original memory has been consolidated^23^. However, the contribution of PL neuronal ensembles encoding fear on long-term memory retrieval is not well understood. Several studies suggest that the PL cortex is engaged during conditioning and is essential for fear expression during memory retrieval. During conditioning, PL neuronal firing correlates with freezing behavior during presentations of the fearful tones^5,6^. Inactivation of the upstream basolateral amygdala decreases PL neuronal tone responses during conditioning^24^ and reduces fear expression upon memory retrieval 24 h later^25^. In addition, PL parvalbumin interneurons decrease firing after FC, which disinhibits PL pyramidal neurons, and induces freezing both before and (2 d) after FC^10^. Together, these studies suggest that PL cortex activity is required for fear expression and is necessary for memory retrieval, at least at early time points (i.e. ≤2 days). Notably, different PL projections drive fear expression after conditioning in a time-dependent manner^26^. Whereas PL neurons projecting to the basolateral amygdala drive fear expression after 6 hours, PL neurons projecting to the paraventricular thalamus are critical after 7 days^11^. Thus, it is likely that distinct PL neuronal ensembles with distinct projection targets gain control over fear expression as the fear memory ages.

Recently, DeNardo *et al*.^20^ investigated whether PL neuronal ensembles TRAPed at different time points after conditioning contribute to subsequent memory retrieval at a remote time point (28 days after conditioning). In this study, the authors employed a new, more efficient TRAP transgenic mouse (TRAP2) and found that PL ensembles TRAPed at 14 days (versus 1 day) after conditioning are more likely to be reactivated during remote (28 d) memory retrieval, and more capable of controlling fear expression^20^. Interestingly, activation of the FC ensemble did not alter tone freezing and had only marginal effects on context freezing (at the 28 d test). Similarly, in our study, we found that chemogenetic inhibition of the FC ensemble in PL cortex did not alter tone freezing (at the 28 d test); however, we also found no effect on context fear. By contrast, a completely different measure of tone fear (conditioned suppression of food seeking) was reduced by this manipulation, perhaps owing to the more sensitive nature of this measure^21^. Collectively, these data suggest that PL cortex FC ensembles participate in distinct modalities of fear expression during long-term memory retrieval, and our study is the first to show that this FC ensemble is capable of regulating remote fear to the CS + (i.e. tone) in particular.

DeNardo *et al*.^20^ also demonstrated that the FC ensemble in PL cortex is critical for recruiting other PL neurons that drive fear expression during remote memory retrieval. To do this, they inhibited PL cortex neurons using a CaMKII-driven Gi-DREADD during FC and found that this manipulation did not alter remote memory retrieval on its own, but precluded their ability to drive context freezing by stimulating a late retrieval (14 d) ensemble during the remote (28 d) memory test^20^. This indicates that fear-encoding (FC) ensembles are necessary to establish other functional ensembles that control remote memory retrieval, even though there may be limited overlap in the neurons comprising FC and remote memory ensembles. This is in line with recent studies showing that PL neurons controlling fear undergo a dynamic functional reorganization during systems memory consolidation that requires distinct inputs to, as well as outputs from, PL cortex^11,27^. However, our study suggests that fear-encoding ensembles in PL cortex directly participate in some forms of CS+ specific fear during remote memory retrieval. This could be due, at least in part, to the nature of the model employed. In this study we used a classical Pavlovian FC model consisting of CS+ shock pairings, while DeNardo *et al*. used a CS+/CS− discrimination model, which may generate both “CS+ danger” and “CS- safety” ensembles in PL cortex^28^. Interestingly, our primary behavioral effect on conditioned suppression of food seeking was attributed exclusively to female rats. This is in line with several clinical and preclinical studies showing that stress-related disorders such as anxiety and PTSD are more prevalent in females^29,30^, as well as recent reports that female rats fail to engage specific stress-buffering PL projections during stressor control^31,32^.

Lastly, it is worth noting that Matos *et al*.^33^ recently demonstrated that the contribution of the FC ensemble in PL cortex to remote memory retrieval may be gated by fear memory strength. These researchers TRAPed the Gi-DREADD in PL cortex after fear conditioning, similar to the approach we used in the present study. However, Matos *et al*. conducted their study in male mice (no females) and used a contextual fear conditioning model (no discrete CS+)^33^. Interestingly, the ability of the TRAP-Gi-DREADD to inhibit context freezing at the remote memory test was contingent on conditioning strength, such that only weak fear memories were subject to regulation. In our study, freezing levels were low in both sexes, suggestive of a weak fear memory trace. Indeed, this could be one reason why we detected an effect on conditioned suppression instead of freezing, since the former is a more sensitive measure of fear^21^. However, it should be noted that females have been shown to exhibit greater conditioned tone freezing than males during extinction (despite no apparent differences during conditioning), and this prolonged expression of learned fear in the absence of danger was associated with sustained activity in the female PL cortex^34^. Because of the different methodologies used between these studies and ours, additional research investigating the influence of fear memory strength between male and female subjects is warranted.

In summary, we demonstrated a contribution of PL cortex of fear-encoding ensembles to remote fear memory retrieval. This contribution was moderate, suggesting that additional brain regions and/or neuronal ensembles also contribute to remote fear memory retrieval. Furthermore, the contribution of the FC ensemble was influenced by sex as well as fear modality. Although the effects we observed on fear measured by conditioned suppression of food seeking could be attributed exclusively to females, additional studies are needed to verify these findings in a larger population. Future studies should also extend these findings to address the mechanisms by which PL neurons encoding fear contribute to the expression of distinct fear behaviors over time. Although it is clear that the PL cortex undergoes a dynamic reorganization during systems consolidation to modulate fear expression after memory retrieval^26^, it is an open question whether the fear-encoding ensemble encompasses all PL cortex projections that dynamically mediate fear memory retrieval over time. This study adds to the growing body of evidence supporting sex differences in PL cortex contributions to fear expression^34^, and are further supported by emerging findings that females do not engage PL cortex-mediated stress-coping strategies^31,32^. Going forward, it will be necessary to examine how the PL cortex ensembles regulating fear expression change over time as the fear memory ages, in males and females separately.

## Methods

All animal procedures followed guidelines approved by the Medical University of South Carolina’s Institutional Animal Care and Use Committee (IACUC). Subjects were age-matched male (n = 17) and female (n = 19) Wistar rats (strain #002, Charles River). Animals were single-housed on a regular light cycle (lights on 7am-7pm) in a temperature and humidity-controlled environment.

### Drug preparation

4-hydroxytamoxifen (4-OHT; Sigma, Cat# H6278) was dissolved at 2 mg/ml in saline with 2% tween-80 and 5% DMSO by careful mixing at room temperature and was prepared fresh the day of use. Rats were injected with 4-OHT (10 mg/kg, i.p.) or vehicle (5 ml/kg, i.p.) two hours after fear conditioning (or no-shock control condition). Clozapine-N-oxide (CNO, NIDA Drug Supply) was dissolved in sterile saline with 5% DMSO at a concentration of 10 mg/ml and used within one week of preparation. All rats were injected with CNO (10 mg/kg, i.p.) 30 min before the remote memory retrieval test. Some rats received an intraperitoneal injection of cocaine at the end of the experiment (cocaine hydrochloride, 10 mg/ml/kg, NIDA Drug Supply) to induce Fos^35^ (30 min after CNO), to verify the inhibitory functionality of the TRAP-Gi-DREADD.

### Virus injections

All rats were injected with a cocktail of virus into the PL cortex containing AAV8-cfos-ER^T2^-Cre-ER^T2^-PEST (1.37 × 10^12^ GC/ml; Stanford Viral Vector Core) and an AAV2-DIO-hM4Di-mCherry (2.3–3.2 × 10^12^ GC/ml; Addgene #44362). For stereotaxic surgery, animals were anesthetized with a cocktail 100 mg/kg ketamine and 7.5 mg/kg xylazine. Virus was injected bilaterally into the PL cortex using the following coordinates: +2.8 mm anterior, ±0.6 mm lateral, and −3.8 mm ventral to Bregma. Rats were allowed to recover for at least a week with food and water available ad libitum before food restriction (20–22 g/day for males; 15–18 g/day for females) and subsequent food training.

### Fear behavior

Rats were trained to lever press for food over ~1 month prior to fear conditioning. Training began on an FR1, and then progressed from an RI-5 to a final RI-60 schedule of reinforcement. Once responding was stable on an RI-60, rats were either fear conditioned (FC groups) or presented with the same number of tones but no shocks (NS group) in the behavioral chamber. Behavioral chambers consisted of a square cage (24 × 30 × 20 cm) with a grid floor wired to a shock generator, positioned inside a sound-attenuating chamber (Med-Associates, Inc). The fear conditioning protocol consisted of 6 tones (white noise, 75 dB, 30 s) that co-terminated with a footshock (1 s, 0.65–0.75 mA), presented on a variable interval of ~3 min for a total session duration of 25 min. During this session and the subsequent retrieval test, rats were allowed to continue lever pressing for food on an RI60 schedule of reinforcement, to provide a baseline of activity against which freezing could be reliably measured in an automated fashion with Ethovision XT tracking software (Noldus Information Technology). At the same time, conditioned suppression of food seeking was measured during each tone presentation, which provides a more sensitive measure of fear than freezing^21^. To analyze suppression data, a suppression ratio was calculated using the following formula: tone press rate/(tone press rate + pretone press rate). This formula produces a ratio of 0.5 when press rate is unaltered by the conditioned stimulus, and <0.5 when pressing is suppressed during tone presentations. Proper acquisition of fear conditioning was defined in all shock groups as freezing >6.1 s during acquisition. This criterion was required to have sufficient power to observe significant acquisition of fear conditioning measured by tone freezing (freezing time >2 standard deviations from baseline freezing). Twenty-eight days after fear conditioning (and TRAPping of fear encoding ensembles), all rats returned to the conditioning chamber for a remote memory retrieval session consisting of 8 tone presentations in the absence of shocks. Tone freezing and suppression ratio were averaged over the 8 tone presentations for statistical analysis. Context freezing was also measured during the 60 s preceding the first tone during the test. Final group sizes represent the pooled results from 3 independently-run behavioral cohorts [NS TRAP+: n = 14 (8 females, 6 males), FC TRAP+: n = 11 (5 females, 6 males), FC TRAP−: n = 11 (6 females, 5 males)]. Animals were assigned randomly to experimental groups, and all groups were run in parallel.

### Histology and immunostaining

Animals were perfused transcardially with saline followed by 10% formalin. Brains were dissected, post-fixed in 10% formalin for 1 h, cryoprotected in 30% sucrose, and stored at −80 °C until sectioning. Serial coronal sections (40 µm) were obtained using a cryostat, collected in chilled 0.1 M PBS containing 1% sodium azide and stored at 4 °C until further processing. For immunohistochemistry, sections were incubated in PBS-Triton X-100 (0.3% PBS-T) with 2% donkey serum (at room temperature for 2 h) and incubated at 4 °C overnight with rabbit anti-cFos (1:2000, Millipore Cat# ABE457, RRID: AB_2631318) and/or chicken anti-mCherry (1:5000, LifeSpan, Cat# LS-C204825, RRID: AB_2716246). Tissue was then incubated for 2 h in 0.3% PBS-T containing fluorescent Alexa-conjugated secondary antibodies (1:500, Jackson ImmunoResearch Labs) and (for some tissue) NeuroTrace fluorescent Nissl (640/660, ThermoFisher). Sections were mounted onto slides and coverslipped with Fluoromount G (Electron Microscopy Sciences). Image stacks were acquired with a Leica SP5 confocal microscope (10x air objective, 1024 × 1024 pixels, 4 frame averages, 1.5x zoom), and imported into Imaris software (Bitplane). The number of Nissl bodies, Fos^+^ and hM4Di-mCherry^+^ neurons in the PL cortex was automatically quantified using the Imaris spot detection function on thresholded images. The number of co-expressing neurons in each section were determined using the colocalize spot function. For all rats, the number of cells was averaged within each hemisphere across 4 sections. Bilateral mCherry expression in the PL cortex was required for inclusion in the final data set. Two rats were eliminated (one for unilateral mCherry expression in the PL cortex, and one for off-target expression in the infralimbic cortex).

### Statistical methods

Behavioral and histological data were obtained in 3 independent cohorts of animals (Cohort 1 = 3 males, 6 females; Cohort 2 = 4 males, 4 females; Cohort 3 = 10 males, 9 females). Ensemble validation and Fos data (Fig. 1) were analyzed with one-way ANOVAs followed by Tukey’s post-hoc comparisons. Freezing and suppression behavior (Fig. 2) were analyzed with one-way or two-way (RM) ANOVAs followed by Tukey’s post-hoc comparisons. Data are graphed as mean ± standard error of the mean. Statistical outliers (n = 2) were identified using the Grubbs method (alpha = 0.05). Significance was defined as alpha = 0.05, and all statistical tests were performed in Prism (GraphPad).

## Supplementary information

Supplementary Materials
