# Supplementary material for: Prefrontal cortex neuronal ensembles encoding fear drive fear expression during long-term memory retrieval
Source: Sci Rep. 2019 Jul 24;9:10709. doi: 10.1038/s41598-019-47095-7 (PMC6656710; doi:10.1038/s41598-019-47095-7)
Supplement: Supplementary file 1 — Supplementary Materials [file 41598_2019_47095_MOESM1_ESM.pdf]

Supplementary materials

Prefrontal cortex neuronal ensembles encoding fear drive fear expression during long-term memory retrieval

Giuseppe Giannotti[1,2], Jasper A. Heinsbroek[1,2], Alexander J. Yue[1], Karl Deisseroth[3,4], and Jamie Peters[1,2*]

[1]Department of Neuroscience, Medical University of South Carolina, Charleston, SC, USA
[2]Department of Anesthesiology, Department of Pharmacology, University of Colorado Denver, Anschutz Medical Campus, Aurora, CO, USA
[3]Department of Bioengineering, CNC Program, Stanford University, Stanford, CA, USA
[4]Department of Psychiatry, Department of Biology, Stanford University, Stanford, CA, USA
*correspondence to: jamie.l.peters@ucdenver.edu

Supplemental Figure 1

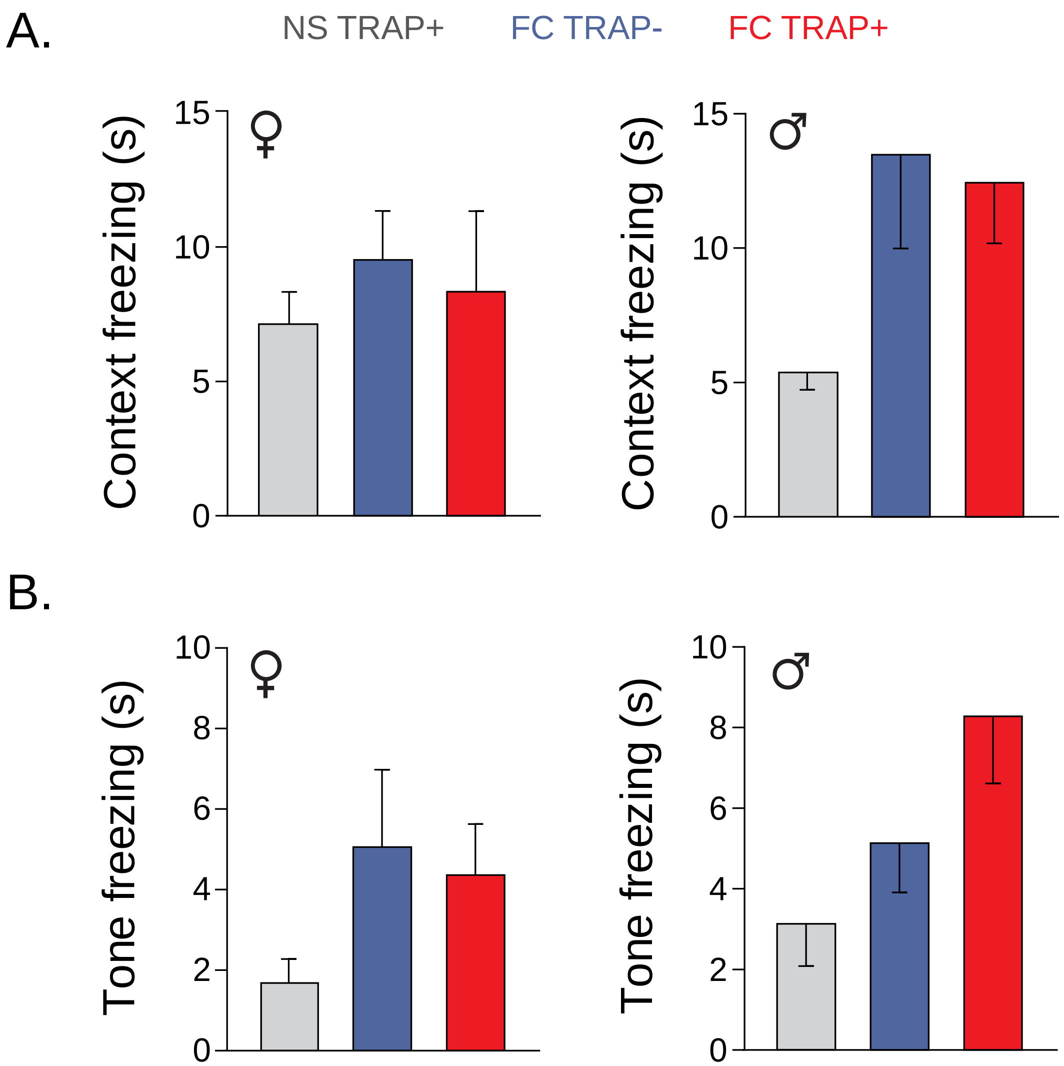

Figure S1. No sex differences were observed in context or tone freezing at test. A. Context freezing was not different between females (left) and males (right) [2-way ANOVA: No main effect of sex or interaction. Main effect of treatment $F(2,30) = 3.618$, $p = 0.0391$]. B. Tone freezing also did not differ by sex [2-way ANOVA: No main effect of sex or interaction. Main effect of treatment $F(2,30) = 4.765$, $p = 0.0160$].
